# 611. Meropenem Dosage Optimization in Critically Ill Patients Based on a Population Pharmacokinetic Approach.

**DOI:** 10.1093/ofid/ofac492.663

**Published:** 2022-12-15

**Authors:** Melissa Romano-Aguilar, Arturo Ortiz-Álvarez, Susanna Medellín-Garibay, Fidel Martínez-Gutiérrez, Helgi Jung-Cook, Rosa C Milán-Segovia, Silvia Romano-Moreno

**Affiliations:** Universidad Autónoma de San Luis Potosí, San Luis Potosí, San Luis Potosi, Mexico; Hospital Central “Dr. Ignacio Morones Prieto”, San Luis Potosi, San Luis Potosi, Mexico; Universidad Autónoma de San Luis Potosí, San Luis Potosi, San Luis Potosi, Mexico; Universidad Autónoma de San Luis Potosí, San Luis Potosí, San Luis Potosi, Mexico; Universidad Nacional Autónoma de México, Mexico city, Distrito Federal, Mexico; Universidad Autónoma de San Luis Potosí, San Luis Potosi, San Luis Potosi, Mexico; Universidad Autónoma de San Luis Potosí, San Luis Potosi, San Luis Potosi, Mexico

## Abstract

**Background:**

Meropenem (MRP) is commonly used to treat serious infections and displays wide variability in plasma concentrations after administration of the same dose in critically ill patients due to factors that affect MRP volume of distribution (V) and clearance (CL) (e.g. edema, sepsis, kidney failure). These alterations could lead to not achieve the pharmacokinetic/pharmacodinamic (PK/PD) target with the consequent failure of antibacterial therapy.

The aim of this study was to describe MRP pharmacokinetic parameters in critically ill patients in order to establish safe and effective initial dosing regimens adapted to patients characteristics.

**Methods:**

This prospective observational study enrolled 78 critically ill patients receiving MRP based on the clinical, biochemical and microbiological findings. Blood sampling occurred at pre-dose, 1, 3 and 6h post-dose. MRP plasma concentrations were determined by high-performance liquid chromatography. Population pharmacokinetic modelling and Monte Carlo simulations were executed with NONMEM. Several regimen dosages of MRP under different scenarios were simulated in order to achieve high probability of target attainment (PTA > 90%) for PK/PD targets of %t > CMI 50% and 100%.

**Figure:**
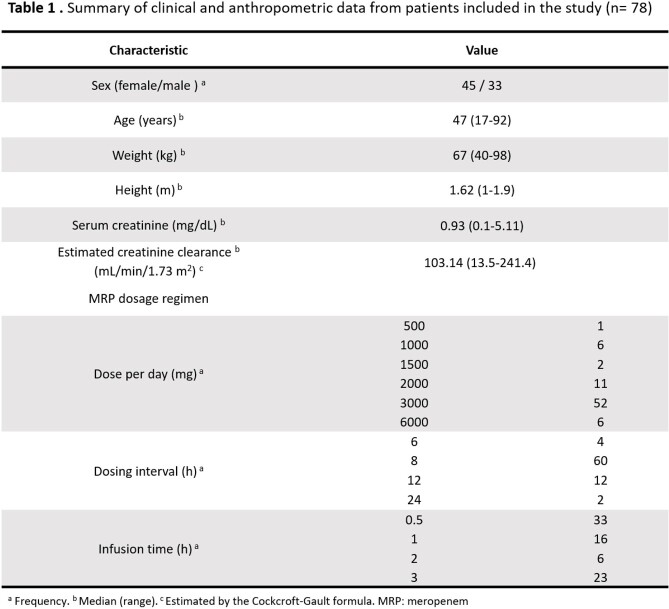

**Results:**

In critically-ill Mexican patients, MRP PK were best described by a one compartment model. The final population model was: *CL (L*/*h*) = 11.9 ∗ (*CLCr*/102.23) and *V (L)* = 25.2. Final model was internally validated proving that it was stable and showed an adequate estimation of variability. Precision and bias fit were assessed through external validation comparing the predictive performance of the base and final models. Different initial dosage regimens were found for CLCr values in which the clinician can choose between a lower dose, a longer dosage interval or a shorter infusion time. However, for patients with augmented renal clearance or PK/PD target 100%t > MIC it was observed that no regimen complied PTA > 90%, suggesting a continuous infusion would be more appropriate.

**Figure:**
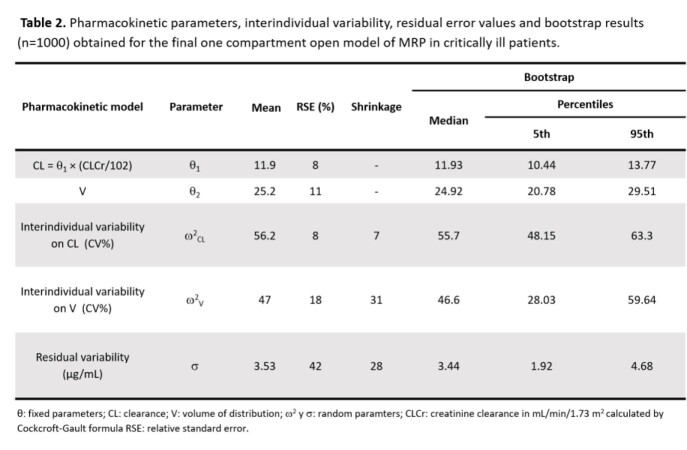

**Figure:**
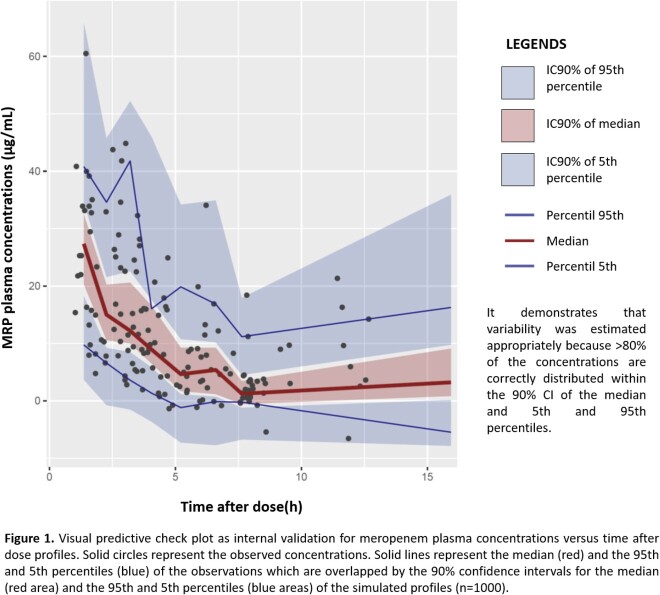

**Figure:**
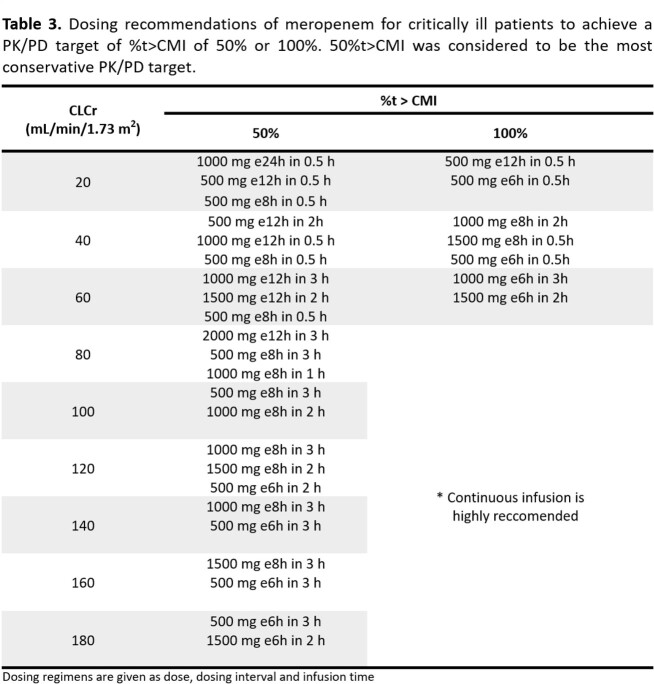

**Conclusion:**

This study demonstrates the wide variability in MRP pharmacokinetics and enhances the need to include therapeutic drug monitoring as part of stewardships interventions in critically ill patients to maximize bacteriological and clinical responses.

**Disclosures:**

**All Authors**: No reported disclosures.

